# Metabolic and inflammatory functions of short-chain fatty acid receptors

**DOI:** 10.1016/j.coemr.2020.06.005

**Published:** 2021-02

**Authors:** Daniele Bolognini, Domonkos Dedeo, Graeme Milligan

**Affiliations:** Centre for Translational Pharmacology, Institute of Molecular, Cell and Systems Biology, College of Medical, Veterinary and Life Sciences, University of Glasgow, United Kingdom

**Keywords:** Short-chain fatty acids, G protein–coupled receptors, FFA2, FFA3, Enteroendocrine, Pancreas, Immune cells, ALDH1A2, aldehyde dehydrogenase 1 family member, BAFF, B-cell activating factor, CMTB, 4-chloro-α-(1-methylethyl)-N-2-thiazolylbenzeneacetamide, DREADD, Designer Receptor Exclusively Activated by Designer Drug, GSIS, glucose-stimulated insulin secretion, GLP-1, glucagon-like peptide 1, GTT, glucose tolerance test, HFD, high-fat diet, IgA, immunoglobulin A, IgG, immunoglobulin G, ILC3, type 3 innate lymphoid cell, KO, knock-out, PA, (S)-2-(4-chlorophenyl)-3,3-dimethyl-N-(5-phenylthiazol-2-yl)butanamide, PNS, peripheral nervous system, PYY, peptide YY, SCA, small carboxylic acid, SCFA, short-chain fatty acid, SCG, superior cervical ganglion

## Abstract

FFA2 and FFA3 are receptors for short-chain fatty acids which are produced in prodigious amounts by fermentation of poorly digested carbohydrates by gut bacteria. Understanding the roles of these receptors in regulating enteroendocrine, metabolic and immune functions has developed with the production and use of novel pharmacological tools and animal models. A complex (patho)physiological scenario is now emerging in which strategic expression of FFA2 and FFA3 in key cell types and selective modulation of their signalling might regulate body weight management, energy homoeostasis and inflammatory disorders.

## Introduction

Since their deorphanisation in 2003 as short-chain fatty acid (SCFA) receptors FFA2 and FFA3 have been viewed as potentially attractive drug targets for the regulation of metabolic and related disorders [5]. However, because they share the same endogenous SCFA ligands and are co-expressed in some tissues, and the development of potent, selective and pan-species active ligands has been frustratingly slow, detailed understanding of their contribution to (patho)physiological conditions has been challenging. Despite this, appreciation that the microbiota produce SCFAs in abundance and that these influence many aspects of the biology of metabolic and immune cell functions has resulted in major efforts, including the development of a number of novel preclinical models, to better understand the roles of both of these G protein–coupled receptors. Herein, we summarise recent insights into the functions of these SCFA receptors in relation to the endocrine, metabolic and inflammatory systems and consider whether they can currently be considered as valid pharmacological targets.

## Roles in enteroendocrine functions

**Figure 1:**
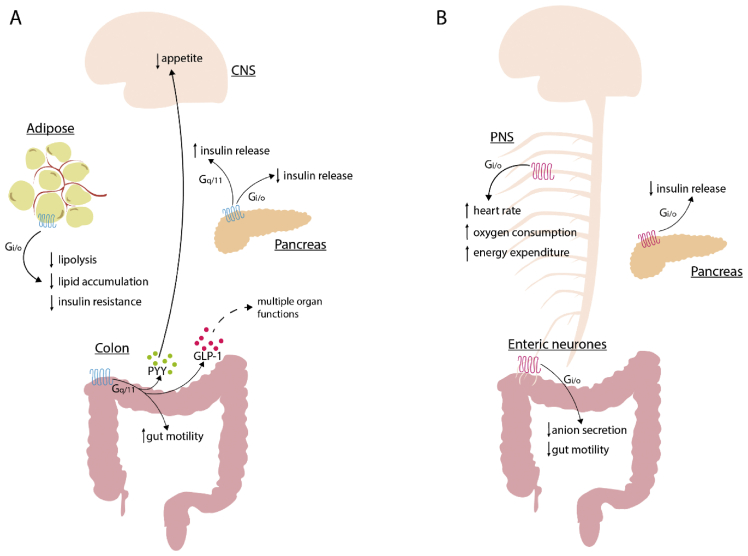
The physiological roles of FFA2 and FFA3. **(a)** SCFA-activated colonic FFA2 triggers increased gut motility and the release of anorectic hormones PYY and GLP-1 from colonic crypts, which in turn decrease appetite by targeting the brain and affect multiple organ functions, respectively. In adipocytes, FFA2 activation inhibits lipolysis, lipid accumulation and lowers insulin resistance in a G_i/o_-dependent manner. Activation of FFA2 in pancreatic beta cells increases or decreases insulin release in a G_q/11_ and G_i/o_-dependent manner, respectively. **(b)** Enteric neuronal FFA3 activation leads to a decrease in anion secretion and gut motility, whereas the activation of FFA3 in pancreas decreases insulin release. FFA3 is expressed in the PNS, where its activation leads to an increased heart rate, oxygen consumption and energy expenditure. All FFA3 functions reported are G_i/o_-mediated. CNS, central nervous system; PNS, peripheral nervous system; GLP-1, glucagon-like peptide 1; PYY, peptide YY; SCFA, short-chain fatty acid. .

Over the years, many reports have clearly shown functions for SCFAs in enteroendocrine hormone release, particularly of the anorectic hormones, glucagon-like peptide 1 (GLP-1) and peptide YY (PYY) from the lower gut of both mouse and human [45,12]. However, direct roles of SCFA receptors, and which might be the major contributor to these functions, has been less certain because both FFA2 and FFA3 are expressed by enteroendocrine L cells at the mRNA [45] and protein level [33]. Studies performed using tissue from conventional mouse knock-out (KO) lines have been helpful, but not definitive. In colonic crypts derived from FFA2 KO mice, the effect of SCFAs on GLP-1 release is absent [45,7,4], whereas in FFA3 KO mice the SCFA effect has been reported to be moderately reduced [45]. By taking a pharmacological route, selective activators of both FFA2 and FFA3 have been reported to induce GLP-1 release from colonic crypts of wild-type mice [33]. However, the ligands used for these studies are allosteric, meaning that they bind at sites distinct from SCFAs, and it is at least possible that although they do indeed activate the relevant receptor they may not generate entirely equivalent signals to those produced by SCFAs. Moreover, although high affinity antagonists for human FFA2 have been described, these have poor if any affinity for the mouse form of FFA2 [36,42]. To attempt to overcome these limitations, Bolognini et al. [6] recently used a chemogenetic approach in which they generated and characterised a knock-in line of transgenic mice where mouse FFA2 was replaced with a modified form of the human FFA2 receptor which, as well as maintaining effective blockade by human selective antagonist drugs, was no longer activated by SCFAs but instead by molecules related to sorbic acid which were shown to have exactly the same signalling profile and capacity as SCFAs at wild type FFA2. Using this Designer Receptor Exclusively Activated by Designer Drug (FFA2-DREADD) concept they showed that activation of the FFA2-DREADD induced GLP-1 release from primary colonic crypts, in colonic explants and an *in vivo* intracolonic stimulation model in which GLP-1 release was sampled from the portal vein [6]. The magnitude of these effects was equivalent to those produced by SCFAs in wild-type mice and were also abolished by the human FFA2 selective antagonists, thus demonstrating the exclusive role of FFA2-mediated GLP-1 release without an obvious contribution from FFA3 (Figure 1). Activation of FFA2 in enteroendocrine cells promotes a G_q/11_-inositol phosphate–calcium signalling pathway [45,33,4,6] that presumably promotes hormone release, although a recent report also suggests a role for an endosomal Gα_i_/p38 signalling pathway in FFA2-mediated GLP-1 release [8]. FFA2 also appears to play an important role in the differentiation of GLP-1^+^ enteroendocrine cells during embryonic stages, where the receptor is detected in the intestinal tract as early as E15.5 [22]. SCFAs may also promote release of the orexigenic peptide Insl5 from a subtype of colonic enteroendocrine L-cell in concert with GLP-1 and PYY [2]. The specific involvement of FFA2 in Insl5 release remains, however, to be fully explored.

## Roles in adipose tissue

FFA2 is highly expressed in adipose tissue, where it appears to be present in both white adipose cells and also in resident macrophages [5,32]. Although FFA3 expression was also initially reported in adipose, later reports have largely discredited this [5]. In adipocytes, activation of FFA2 inhibits lipolysis [4]; Figure 1. This was recently re-assessed using the FFA2-DREADD mouse model described earlier. Here, the FFA2-DREADD agonist sorbic acid inhibited beta-adrenergic–stimulated lipolysis in a concentration-dependent and pertussis toxin (PTX)–sensitive manner, indicating that activation of G_i_ signalling pathways were required [6]. It is interesting that although in cell line-based studies FFA2 can activate a diverse range of heterotrimeric G proteins [6], in physiological settings different signalling mechanisms are used in different cell types to specify function because, as noted earlier, FFA2-mediated GLP-1 release reflects activation of G_q/11_-linked signalling. The antilipolytic effect of FFA2 activation suggests an opportunity to target this receptor in weight management with consequent implications for type 2 diabetes (Figure 1). However, this subject is complex. Earlier studies suggested a negative role of FFA2 on weight and metabolic parameters in mice fed a high-fat diet (HFD) [3], whereas Kimura et al. [23] (2013) noted that FFA2 KO mice fed a HFD had a detrimental effect on fat accumulation and body weight, but also on glucose tolerance and energy expenditure, effects that were counterbalanced in mice selectively overexpressing FFA2 in adipocytes. Such outcomes were recently supported by the same group using a HFD-fed wild-type mouse model in which animals were treated with GCL2505, a probiotic bacterial strain able to promote production of SCFAs in the gut. Here, GCL2505 was found to increase energy expenditure with a resultant decrease in fat accumulation and improvement in insulin sensitivity, effects that were absent in equivalently treated FFA2 KO mice [17]. The basis for the different outcomes reported is unclear but may reflect differences in the genetic background of the animals and the strategies used to target FFA2 expression.

A role of FFA2 in energy metabolism in mice has recently also been shown under ketogenic conditions, and the ketone body acetoacetate proposed as a novel additional endogenous ligand for FFA2 [31]. In wild-type mice subject to starvation or a ketogenic diet, a contribution of an acetoacetate-FFA2 axis in weight management, fat accumulation and energy expenditure was postulated. Moreover, FFA2 KO mice were reported to have decreased levels of lipoprotein lipase in adipose and liver tissues and hence decreased control of lipid metabolism.

Although these rodent-based studies are of interest, it is vital that human studies might confirm positive effects of SCFAs on energy homoeostasis and potentially explore if these reflect FFA2 receptor activation. Rectal administration of SCFAs to obese individuals in a randomised crossover trial showed an increase in fasting lipid oxidation and resting energy expenditure, parameters that were positively correlated with an increase in plasma levels of the SCFA acetate. In addition, a decrease in whole body lipolysis was reported, supporting effects of SCFAs on adipose tissue [9], potentially via activation of FFA2. Further studies have evaluated acute effects of propionate administered orally to healthy volunteers. Here, propionate increased resting energy expenditure in overnight-fasted subjects, an effect that was independent of changes in glucose and insulin levels [10]. The authors found an increase in whole body lipid oxidation, further strengthening previous studies conducted in human and murine models. In addition, provision of inulin propionate ester to nondiabetic overweight or obese individuals significantly improved adipose tissue insulin resistance compared with treatment with inulin alone [11]. Although it is tempting to speculate that the effects of the SCFAs in these studies might reflect activation of a SCFA receptor, potentially FFA2, by their nature these studies were not designed to address this question directly.

## Roles in pancreas

The physiological functions of SCFA receptors in pancreas are rather controversial. Although both receptors are expressed in beta cells, there are currently opposing data in regard to the effect of SCFAs. Tang et al. [44] reported an inhibitory effect of acetate on glucose-stimulated insulin secretion (GSIS) on isolated mouse and human islets, whereas more recently SCFAs were indicated to have little or no effect in an isolated pancreas infusion model [34]. The opposite was observed in perifused murine and human islets where both acetate and propionate increased GSIS [35]. While this may reflect the expression of multiple targets for SCFA action, the difference in experimental procedures adopted to measure insulin release is also a plausible explanation.

The diverse signalling pathways downstream of these receptors should also be considered. Although FFA3 signalling in pancreatic beta cells is G_i_-linked [38], FFA2 appears to couple to both G_i_ and G_q/11_ pathways consistent with its promiscuous capacity to activate multiple G protein families in transfected cell–based studies [29,39]. Generally, G_i_ signalling in beta cells is considered to inhibit insulin release, whereas G_q/11_ signalling promotes this [4]. Accordingly, incubation of wild-type–derived islets with either of a pair of FFA2-selective small carboxylic acid ligands described originally by Schmidt et al. [40], small carboxylic acid 14 and 15 caused an increase in GSIS via G_q/11_-dependent phospholipase C (PLC) activation, whereas incubation with the FFA2 allosteric agonist ligand 4-chloro-α-(1-methylethyl)-N-2-thiazolylbenzeneacetamide caused a decrease in GSIS in a PTX-sensitive and therefore G_i_-mediated manner [39]. Similarly, activation of FFA2 with the related allosteric agonist (S)-2-(4-chlorophenyl)-3,3-dimethyl-N-(5-phenylthiazol-2-yl)butanamide, but not acetate, was found to potentiate GSIS in both mouse- and human-derived islets in a PTX-insensitive manner [29]. It is plausible that selective activation of FFA2 signalling pathways results in opposing outcomes, and this should now be fully characterised using known FFA2-selective biased ligands such as AZ1729 [5]; Figure 1. Recently, a key role of FFA2 was proposed for the ability of *Leuconostoc mesenteroides* EH-1–derived butyric acid to increase insulin and decrease plasma glucose levels in a murine model of type 1 diabetes [47]. The potential contribution of FFA2 was defined based on a blockade by the antagonist GLPG0974. However, it is known that although GLPG0974 is a high affinity antagonist for human FFA2, as noted earlier it lacks affinity for murine FFA2 [42]. As such, any effect of GLPG0974 in mouse models must reflect off-target effects that are unrelated to FFA2. Results such as those reported by Traisaeng et al. [47] must therefore be interpreted with caution and such conclusions certainly require validation using other pharmacological or genetic tools. FFA2 is also expressed in the pancreas at embryonic stages [22]. Here, a SCFA-FFA2 axis appears to control insulin secretion, with FFA2 KO embryos showing reduced insulin and higher plasma glucose levels compared with the wild type [22]. With regard to FFA3, data from transgenic mouse models suggest a negative contribution of this receptor on insulin release (Figure 1). Even though islets derived from FFA3 KO mice showed higher levels of insulin than the wild type when stimulated with high concentrations of glucose, the opposite was found in islets isolated from mice selectively overexpressing FFA3 in pancreatic beta cells [48]. Similarly, in an *in vivo* glucose tolerance test FFA3 KO mice showed lower glucose levels than the wild type, whereas the opposite was observed in FFA3-overexpressing mice [48]. Others have recently supported such outcomes using a hyperglycaemic clamp model, with FFA3 KO mice showing an improved insulin profile compared with wild-type animals [37]. Although FFA3 activation appears to be detrimental for insulin release, no alteration in insulin sensitivity has been detected in FFA3 KO animal models [48,37]; Figure 1.

In human trials, both fasting and postprandial glucose and insulin profiles were found to be unchanged after colonic infusion of SCFAs to overweight/obese male subjects [9]. Similar data were obtained by acute oral administration of propionate in fasted human subjects [10]. Again, whether this is the result of FFA2 and FFA3 producing opposing effects in the pancreas is yet to be fully defined. However, considering that both receptors seem functional in this organ, selective inhibition of FFA3 and/or activation of the FFA2-dependent G_q/11_ pathway might provide a key to selectively stimulate insulin release.

## Roles in the peripheral nervous system

Undoubtedly FFA3 is expressed in peripheral sympathetic neurons as suggested initially by detection at both mRNA [21] and protein levels [33,14], where the receptor appears to be localised in a heterogeneous population of cells. Kimura et al. [21] showed FFA3 expression and excitatory activity in superior cervical ganglia neurons. Elimination of the receptor in mice had a negative effect on heart rate and oxygen consumption, suggesting a role in energy expenditure [21]; Figure 1. The same group recently confirmed the expression, and physiological importance of FFA3 in the sympathetic ganglia also at the embryonic stage, where the receptor appears to play a role in sympathetic nerve differentiation. Here, they observed a decrease of innervation of the heart, heart rate, body temperature and oxygen consumption in FFA3 KO embryos, suggesting a contribution of a SCFA-FFA3 axis in energy metabolism [22]; Figure 1. These effects were also present in offspring derived from mothers treated with antibiotics to dampen SCFA levels, but reversed by treatment with propionate during pregnancy [22].

Detection of the FFA3 receptor has been shown in both superior cervical ganglia and coeliac-superior mesenteric ganglion neurons in a transgenic mouse model in which red fluorescent protein is expressed from the FFA3 receptor promoter [14]. In both neuronal populations, FFA3 activation had an inhibitory effect on N-type calcium channel function [14]. However, its physiological significance is yet to be determined and probably depends on the neuronal subtypes expressing FFA3, with the majority being noradrenergic and vasoconstrictor in nature [14]. Other work has focused on FFA3 expression in the enteric nervous system. Nohr et al.[33] showed the presence of FFA3 in neurons of the mucosal, submucosal and myenteric plexus in the small intestine (Figure 1). Similar expression patterns have been shown in the rat proximal colon, where activation of the receptor inhibits cholinergic-mediated anion secretion [20,19]. In another report, FFA3 activation in distal regions of mouse intestine caused a reduction of short–circuit currents in a tetrodotoxin-sensitive manner, an effect that was also replicated in human colonic tissues [46]. Although FFA3 expression in cholinergic neurons of the intestinal mucosa and submucosal layer might play a role in intestinal secretion [20], FFA3 in the myenteric plexus might instead play a role in intestinal contraction and motility [19]. Acute activation of FFA3 has been shown to block serotonin-induced intestinal motility [19], but pharmacological tools to selectively activate FFA3 are even more limited than for FFA2 [30], restricting interpretation. Similarly, a decrease in gut motility has been observed *in vitro* using mouse colonic preparations acutely treated with potentially selective FFA2 and FFA3 agonists [46]. However, Bolognini et al. [6] failed to observe a contribution of FFA3 to gastric motility when hFFA2-DREADD mice, in which SCFAs such as propionate are unable to activate the modified FFA2 receptor, were provided with propionate in the drinking water for 5 days. Whether acute versus chronic administration of SCFAs might have differential impacts on intestinal motility warrants further investigation.

## Immune functions

**Figure 2:**
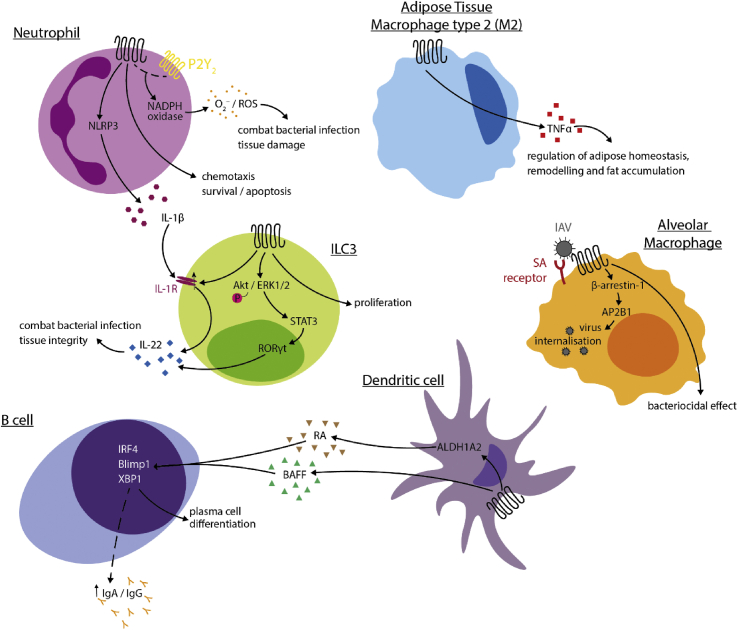
Roles of FFA2 in immune cell populations. In neutrophils, activation of FFA2 triggers chemotaxis and potentially promotes neutrophil survival or apoptosis, promotes superoxide production (in crosstalk with P2Y2R) and the production of the cytokine IL-1β. Complementing this action, in ILC3 cells, activation of FFA2 leads to an upregulation of IL-1R, which is in turn activated by IL-1β to produce IL-22. FFA2 activation also directly leads to IL-22 production via the Akt/ERK1/2- signal transducer and activator of transcription 3– RAR-related orphan receptor γ t axis in these cells. In dendritic cells, activation leads to the production of BAFF and A2ALD1a2, the latter of which catalyses the production of retinoic acid, which along with BAFF promotes B-cell differentiation into plasma cells. FFA2 may also be involved in IgA/IgG release via an unknown mechanism. Activation of FFA2 in type 2 macrophages promotes the release of TNFα, as well as bacteriocidal activity through an unknown pathway. FFA2 may also function as a coreceptor for the influenza A virus, triggering a β-arrestin-1-dependent internalisation of the virus. AKT, protein kinase B; ALDH1A2, aldehyde dehydrogenase 1 family, member A2; AP2B1, AP-2 complex subunit beta; BAFF, B-cell activating factor; Blimp1, B-lymphocyte-induced maturation protein-1; ERK, extracellular signal–regulated kinase; IAV, influenza A virus; IgA, immunoglobulin A; IgG, immunoglobulin G; IL-1β, interleukin 1β; IL-22, interleukin-22; ILC3, type 3 innate lymphoid cells; IRF4, interferon regulatory factor 4; NADPH, nicotinamide adenine dinucleotide phosphate; NLRP3, NOD-, LRR- and pyrin domain-containing protein 3; P2Y2, P2Y purinoreceptor 2; RA, retinoic acid; ROS, reactive oxygen species; TNFα, tumour necrosis factor α; STAT3, signal transducer and activator of transcription 3; RORγt, RAR-related orphan receptor γ t; ROS, reactive oxygen species; SA, sialic acid; XBP1, X-box binding protein 1.

There has been extensive research on the role of SCFA receptors in immunity. They, and especially FFA2, may contribute to immune homoeostasis, tissue integrity and responses to pathogens. In mouse, mRNA encoding FFA2 is widely expressed by immune cells and the receptor seems to play roles in inflammatory tissue processes associated with metabolic disorders. For example, FFA2 expression has been reported on M_2_-type macrophages in isolated adipose tissue and stimulation of the receptor is associated with the induction of TNFα, a pro-inflammatory cytokine implicated in adipose tissue homoeostasis, remodelling and fat accumulation. This may contribute to the roles of FFA2 in energy homoeostasis [32]; Figure 2. Undoubtedly, FFA2 is highly expressed in neutrophils, including both blood- and bone marrow–derived cells (immunological genome project database (*http://www.immgen.org/databrowser/index.html*), whereas FFA3 is all but absent. In neutrophils, FFA2 is involved in a variety of cellular processes. FFA2 promotes chemotaxis of isolated mouse and human neutrophils [28,4], resulting in neutrophil recruitment to the site of inflammation or infection, as well as survival of these cells [15]. Other studies conclude that FFA2 plays a role in the resolution of inflammation, leading to neutrophil apoptosis at infection sites, as mice lacking the receptor were found to be more susceptible to *Klebsiella pneumoniae* infection, with increased bacterial proliferation and uncontrolled inflammatory responses [16]. Neutrophil activation results in the formation of reactive oxygen species via FFA2 (Figure 2). Although SCFAs in isolation are not highly effective, co-addition of the types of allosteric activators described earlier, including AZ1729 [4] and compounds of the phenylacetamide class [24,43,30], were recently shown to enhance SCFAs, resulting in potent activation of NADPH-oxidase assembly [27,26]. In addition, crosstalk of FFA2 with the ATP-sensing P2Y_2_ receptor has also been found to stimulate NADPH-oxidase assembly, and thus superoxide production [25]. This may have mixed outcomes: although superoxide is effective in fighting bacterial pathogens, it is also associated with tissue damage in inflammatory responses. Recently, FFA2 expression has been reported in type 3 innate lymphoid cells (ILC3s), including retinoic acid receptor (RAR)-related orphan receptor γ t–positive cells in mouse intestinal tissues, where the modulation of the receptor leads to augmented production and release of IL-22, an important regulator of tissue integrity [13]; Figure 2). This has also been confirmed in another report in which a crosstalk between FFA2 receptor expression in neutrophils and ILC3s has emerged, resulting in a protective effect against a *Clostridium difficile* infection in a mouse model [15]. The interplay between the pro- and anti-inflammatory effects of FFA2 activation on neutrophils and ILC3s highlights the complexity of the involvement of this receptor in the maintenance and resolution of the inflammatory response in disease. Moreover, recent studies have indicated key roles for SCFAs in limiting concomitant pneumococcal infection during influenza A virus infection and that this was likely mediated via FFA2 [41]. However, a distinct report has suggested that FFA2 may act as a coreceptor for influenza A virus entry [49]. Interestingly, as treatment with phenylacetamide-based allosteric agonists restricted influenza A virus entry and this potentially removed FFA2 from the cell surface, then prophylactic enhancement of SCFA levels or the rapid use of FFA2 selective agonists might usefully both limit influenza A entry and replication and limit subsequent bacterial, for example, pneumococcal, superinfection. Similarly, viral load and pulmonary inflammation induced by respiratory syncytial virus infection have been reported to be reduced in mice by provision of a high fibre diet and hence acetate, and that this is defective in animals lacking FFA2 [1].

SCFA receptors have also been implicated in antibody responses by inducing plasma cell differentiation. Dendritic cell FFA2 activation leads to the release of the cytokine B-cell activating factor (BAFF) and the retinoic acid–regulating protein aldehyde dehydrogenase 1 family, member ALDH1A2, which in turn trigger the expression of plasma cell differentiation genes with consequent production of immunoglobulin A and G (IgA and IgG, respectively) [50,51], that are involved in intestinal integrity and immune homoeostasis (Figure 2). Another report recently excluded the implication of FFA2 in butyrate-induced IgA production in mouse large intestine [18]. Whether FFA2 plays distinct roles in small rather than large intestine is yet to be determined.

## Conclusions

A new scenario is emerging in which receptors for SCFAs control energy homoeostasis and metabolic functions through different mechanisms and the contribution of key tissues/cell types expressing these receptors (Figure 1). Hence, SCFA receptors might contribute to the maintenance of energy homoeostasis in a steady state, but their pharmacological modulation might also underpin a novel strategy for the treatment of metabolic and inflammatory disorders. Considering that FFA2 is expressed in multiple tissues and that the receptor appears to favour specific downstream signalling pathways in different settings, then ‘biased’ ligands at this receptor might offer a feasible approach to specifically activate subsets of pathways. For example, an FFA2-G_i_ selective bias agonist might mainly target adipose tissue and improve insulin resistance, whereas an FFA2-G_q/11_ bias agonist might improve the release of anorectic hormones in the intestine and increase insulin release in the pancreas. Although this has not been explored in a systematic manner, Bolognini et al. [4] identified and characterised AZ1729 as such an FFA2-G_i_ selective ligand after initial studies indicating that it lacked activity in Ca^2+^ elevation–based assays. Ideas that activators of FFA2 may be effective in limiting viral load and subsequent bacterial infections are of course intriguing in relation to the COVID-19 pandemic. Understanding of the roles of FFA3 is emerging but lags behind FFA2. However, selective expression in specific cell types in the pancreas and PNS might offer potential for novel treatments.

## Conflict of interest

Nothing declared.
